# Feasibility of a mindfulness‐based weight loss program in an industry setting: A pilot study

**DOI:** 10.1002/pcn5.70265

**Published:** 2025-12-09

**Authors:** Daisuke Yamaichi, Kiwako Yokoyama, Hiroyuki Uchida, Daisuke Fujisawa

**Affiliations:** ^1^ Department of Neuropsychiatry Keio University School of Medicine Tokyo Japan; ^2^ Division of Quality Assurance Programs National Cancer Center Institute for Cancer Control Tokyo Japan

**Keywords:** metabolic syndrome, mindfulness‐based intervention, obesity, occupational health, weight management

## Abstract

**Aim:**

This pilot study evaluated the feasibility, safety, and preliminary effectiveness of a mindfulness‐based weight loss program—Mindful Eating and Lifestyle (MEAL)—implemented in an occupational setting in Japan.

**Methods:**

This retrospective study was conducted between July 31, 2019, and May 31, 2022, at a branch office of a manufacturing company. Of 5016 employees who received annual health check‐ups in 2019, 632 met the company's occupational health criteria for weight management and were informed of the MEAL program. Eleven employees who participated in the MEAL program and provided written informed consent were included in this retrospective study. The MEAL program consisted of four 60‐min group sessions over 2 months, incorporating third‐wave cognitive behavioral therapy (CBT) strategies. Sessions included guided mindfulness practices (breathing and mindful eating to notice hunger, satiety, and emotions) and promoted flexible “quality over quantity” eating. Data on program completion, safety, and body weight were extracted from routine health records with prior informed consent. Paired *t*‐tests were used to analyze weight change among participants with complete data.

**Results:**

All 11 participants completed all four sessions, yielding a 100% completion rate. No adverse events were reported. Among the seven participants with complete weight data, a significant reduction in body weight was observed: from 76.1 kg (SD = 19.6) to 74.8 kg (SD = 19.4), with a mean difference of −1.4 kg (95% CI: −1.9 to −0.8; *p* = 0.001).

**Conclusion:**

The MEAL program demonstrated high feasibility, safety, and potential short‐term effectiveness in a real‐world workplace setting. Further research is needed to confirm long‐term outcomes and generalizability.

## INTRODUCTION

Obesity is a global public health challenge linked to increased risks of cardiometabolic diseases and certain cancers, contributing to premature mortality.^1^, ^2^, ^3^ The prevalence of obesity continues to rise worldwide, underscoring the need for effective, sustainable interventions.^4^, ^5^, ^6^ Even a modest 5%–10% reduction in body weight can significantly improve metabolic outcomes, such as glycemic control and blood pressure, and reduce mortality risk in individuals with overweight or obesity.^7^, ^8^ However, long‐term weight management remains challenging; many individuals regain lost weight over time. Traditional diet and exercise programs show high short‐term efficacy but poor maintenance, partly because they often fail to address underlying psychological factors (e.g., emotional eating, stress‐driven cravings, and deficits in self‐regulation) that precipitate weight regain.^9^ Indeed, maladaptive eating behaviors such as binge eating, emotional eating, and external cue‐driven eating are associated with relapse after weight loss. These observations highlight the importance of approaches that target the psychological determinants of obesity in order to support sustained healthy behaviors.^10^, ^11^

In recent years, “third‐wave” cognitive behavioral therapies (3wCBTs)^12^, ^13^, ^14^ have gained attention as promising strategies for weight management that explicitly tackle these psychosocial factors. 3wCBT approaches emphasize acceptance of internal experiences such as hunger, cravings, or negative emotions rather than avoidance and encourage behaviors aligned with one's personal values and long‐term goals.^15^ By strengthening psychological flexibility and addressing barriers like low self‐efficacy, depression, anxiety, and stress, 3wCBT may facilitate more sustainable weight control compared to traditional behavioral counseling.^16^, ^17^ While mindfulness‐based interventions (MBIs) primarily cultivate present‐moment, nonjudgmental awareness to reduce automatic or emotionally driven eating, acceptance and commitment therapy (ACT) extends this approach by incorporating acceptance and value‐based behavioral commitment.^10^, ^18^ Through this process, ACT aims to enhance psychological flexibility, promoting consistent, goal‐directed behavior even in the presence of discomfort or cravings. For example, ACT, which is a form of 3wCBT, teaches individuals to accept urges or discomfort and commit to value‐consistent actions; this process has been shown to improve coping with food cravings and enhance self‐regulation of eating impulses.^16^ Mindfulness‐based techniques, similarly, cultivate nonjudgmental awareness of the present moment, helping individuals recognize and tolerate internal triggers for overeating. Mindful eating, a core component of many 3wCBT programs, encourages slow, attentive eating and focus on the sensory experience of food, which can heighten satiety recognition and reduce automatic, habitual overeating.^18^ Such strategies interrupt cycles of restrictive dieting and emotional overeating, and help reframe one's relationship with food. From a psychiatric perspective, these interventions are particularly relevant, since they target self‐regulatory capacity and emotional coping skills. Mindfulness training has demonstrated benefits in reducing chronic stress and anxiety, improving stress‐coping patterns, and ultimately may mitigate stress‐related weight gain.^19^, ^20^ It can also decrease episodes of emotional eating by enabling individuals to observe stressors and negative emotions without reacting with food, instead substituting more adaptive behaviors.^10^, ^17^, ^21^

Accumulating research evidence supports the efficacy of 3wCBT approaches in obesity management. Several recent systematic reviews and meta‐analyses indicate that mindfulness‐ and acceptance‐based interventions can improve eating behaviors and yield clinically meaningful weight outcomes.^16^, ^17^, ^22^, ^23^, ^24^, ^25^ These therapies appear to be especially effective in curbing maladaptive eating such as binge or impulsive eating and enhancing psychological well‐being, which in turn supports adherence to healthy lifestyle changes. Collectively, such findings suggest that mindfulness‐oriented interventions can confer enduring benefits for weight regulation and metabolic health by targeting the mind–body aspects of eating. Nevertheless, it should be acknowledged that evidence on very long‐term outcomes remains limited. Few studies have extended beyond 12‐month follow‐ups, and not all have found significant weight loss maintenance, indicating a need for further research to confirm and refine the long‐range effects of these interventions.^10^, ^17^ Additionally, most trials to date have been conducted in Western clinical or community settings; there is a relative paucity of data from non‐Western populations and real‐world contexts such as workplaces. In Japan, for example, mindfulness‐based weight management programs have seldom been formally evaluated, with only a few case reports on mindfulness‐based cognitive therapy (MBCT) in obesity^9^ and a single pilot randomized‐controlled trial involving a mindfulness mobile application for individuals with metabolic syndrome.^26^ This gap in the literature highlights the importance of investigating the applicability and effectiveness of 3wCBT approaches across diverse cultural and occupational settings.

We therefore conducted a pilot study to examine the feasibility and effectiveness of a mindfulness‐based weight loss program in an occupational setting. Specifically, we implemented a brief group intervention, the Mindful Eating and Lifestyle (MEAL) program, grounded in 3wCBT principles, for employees with overweight or obesity at a Japanese company. We then retrospectively assessed implementation indicators, such as session completion and safety, and evaluated pre‐ and post‐intervention changes in weight and related health outcomes.

## METHODS

### Study design and participants

This retrospective study aimed to examine the feasibility of the MEAL program in an occupational setting. The protocol was reviewed and approved by the Keio University School of Medicine ethics committee (approval number 20190099), and all participants provided written informed consent for the use of their health data. The study was conducted at a branch office of an electrical manufacturing company in Japan. As mandated by the national Industrial Safety and Health Law, the company offered annual health check‐ups and follow‐up health guidance for employees at risk of metabolic syndrome. Eligible employees for weight management guidance were defined by the company's occupational health criteria as follows: employees aged ≥ 40 years with body mass index (BMI) ≥ 25 kg/m² and/or clinical risk factors (i.e., HbA1c ≥ 6.5% or waist circumference ≥ 85 cm in men or ≥90 cm in women) and employees aged 18–39 years with BMI ≥ 25 kg/m².

In 2019, all employees meeting these criteria were offered the opportunity to enroll in the MEAL program as part of routine health guidance, and participation was voluntary. The first author (a board‐certified psychiatrist and occupational physician) implemented the program on‐site between July and August 2019 for those who enrolled. Post‐intervention body weight was measured immediately after the final MEAL session. In 2021, all individuals who had participated in the 2019 program received a written explanation of this study, and written informed consent was obtained from those who agreed to the retrospective use of their health‐related data. Two types of data were extracted: (1) baseline characteristics obtained from the 2019 annual health check‐up records and (2) pre‐ and post‐intervention data collected by the company's health support office as part of routine health guidance. The latter included body weight measurements and notes related to safety and program adherence. During the study period, participants did not receive any additional structured interventions for weight management or psychological support.

### MEAL program

The MEAL program was a structured 3wCBT intervention designed to improve dietary habits, self‐regulation, and stress management for weight control. The program consisted of four sessions, each 60 min long, delivered biweekly over approximately 2 months. All sessions were conducted in small groups at the workplace. The content and format of MEAL were developed with reference to previous mindfulness‐ and ACT‐based weight loss interventions, adapting evidence‐based techniques to a concise workshop suitable for busy employees.^18^, ^27^, ^28^, ^29^ Each session combined a brief didactic lecture with interactive exercises and group discussions. Key topics included (a) psychoeducation about obesity, nutrition, and eating behaviors; (b) training in mindfulness meditation and mindful eating practices; (c) stress management skills (recognizing triggers and using mindfulness or breathing techniques to cope with stress); and (d) principles of behavior change drawn from applied behavior analysis (e.g., identifying environmental cues to overeating and learning communication or problem‐solving strategies to support healthy habits). Participants engaged in guided mindfulness exercises, such as mindful breathing and savoring a small portion of food with full attention to cultivate awareness of hunger, satiety, and emotional states during eating. They were encouraged to practice these mindfulness and mindful eating techniques in their daily lives between sessions.

Although no formal exercise regimen was included in the MEAL program, participants were advised to maintain general physical activity of their choosing, such as walking or other enjoyable exercises. The program emphasized “quality over quantity” in eating, aiming to cultivate satisfaction through balanced and enjoyable meals rather than calorie restriction. Instead of prescribing specific foods or calorie limits, participants were encouraged to “eat only what was truly needed, when it was needed,” fostering a sense of satisfaction rather than restraint. Through this process, participants were guided to bring mindful awareness to their eating behaviors and to reflect on whether their cravings represented genuine physical hunger or emotional cues. The program specifically aimed to reduce automatic or cue‐driven eating elicited by internal factors such as stress, boredom, or loneliness, as well as by external stimuli such as advertisements or social situations. Participants were trained to discern whether their sense of hunger was physiological or psychological and to adopt alternative, non‐food strategies for emotional satisfaction. By prioritizing the cultivation of satisfaction over restriction, this approach sought to lower psychological resistance to behavioral change and promote sustainable improvements in eating habits and long‐term self‐regulation.

All sessions conducted in 2019 were led by a psychiatrist trained in mindfulness‐based stress reduction (MBSR) and MBCT, with over 1 year of personal experience in mindfulness practice. This ensured that the therapeutic components, including meditation guidance and group facilitation, were delivered competently and safely.

### Statistical analysis

The primary outcome was a completion rate (i.e., proportion of participants who attended all sessions), representing the feasibility of the MEAL program. Any adverse events and body weight were also assessed. Baseline characteristics were summarized as means ± standard deviations for continuous variables and as frequencies (percentages) for categorical variables. For each outcome, pre‐ and post‐intervention values were compared using paired *t*‐tests. Paired *t*‐tests were used to estimate the mean differences and 95% confidence intervals (CIs), assessing the magnitude and direction of change. Statistical significance was defined as a two‐tailed *p* < 0.05. All analyses were conducted using standard statistical software (IBM spss, version 28).

## RESULTS

Of the 5016 employees who underwent annual health check‐ups in 2019, 632 met the eligibility criteria for weight management guidance based on the company's occupational health standards and were informed about the program. Eleven of them participated in the MEAL program and subsequently provided informed consent for inclusion in this study. All 11 participants completed all four sessions, resulting in a completion rate of 100%. No adverse events were reported during the intervention. Therefore, feasibility and safety outcomes were evaluated using data from all 11 participants.

For the exploratory outcome of body weight change, data from seven participants were analyzed, as complete pre‐ and post‐intervention weight records were available only for these individuals. Baseline characteristics of the seven MEAL participants are summarized in Table 1. A significant reduction in body weight was observed following the MEAL program; the mean body weight decreased from 76.1 kg (SD = 19.6) to 74.8 kg (SD = 19.4), with a mean difference of −1.4 kg (95% CI: −1.9 to −0.8; *p* = 0.001).

**Table 1:** Baseline characteristics of Mindful Eating and Lifestyle (MEAL) participants (*N* = 7).

Variable	Mean ± SD
Age (years)	49.9 ± 10.9
Gender (women, %)	5 (71.4%)
Height (cm)	160.4 ± 12.0
Weight (kg)	76.1 ± 19.6
Body mass index (kg/m²)	29.4 ± 5.5
Waist circumference (cm)	93.2 ± 13.5
Systolic BP (mmHg)	116.4 ± 10.9
Diastolic BP (mmHg)	73.4 ± 8.2
Hemoglobin A1c (%)	6.0 ± 0.6
Uric acid (mg/dL)	6.0 ± 1.3
LDL‐C (mg/dL)	123.3 ± 25.6
HDL‐C (mg/dL)	56.7 ± 10.8
Total cholesterol (mg/dL)	200.6 ± 30.7
Triglycerides (mg/dL)	129.3 ± 106.5
γ‐GTP (IU/L)	46.1 ± 54.5
AST (IU/L)	29.7 ± 14.9
ALT (IU/L)	49.6 ± 43.9
Creatinine (mg/dL)	0.71 ± 0.20
eGFR (mL/min/1.73 m²)	78.3 ± 16.1

Abbreviations: γ‐GTP, gamma‐glutamyl transpeptidase; ALT, alanine aminotransferase; AST, aspartate aminotransferase; BP, blood pressure; eGFR, estimated glomerular filtration rate; HDL‐C, high‐density lipoprotein cholesterol; LDL‐C, low‐density lipoprotein cholesterol.

## DISCUSSION

This study aimed to evaluate the feasibility, safety, and preliminary effectiveness of the MEAL program, a four‐session group‐based weight loss intervention implemented in a real‐world occupational setting. All participants completed the program without adverse events, and a significant reduction in body weight was observed among those with complete pre‐ and post‐intervention data.

The MEAL program demonstrated high feasibility, as all 11 participants attended all four sessions without dropout, resulting in a 100% session attendance rate, and no adverse events were reported. However, post‐intervention body weight data were missing for four participants in the company's health records. In addition, participants achieved a statistically significant weight loss of 1.4 kg, corresponding to a mean relative reduction of −1.8% in body weight. This magnitude of change, while modest, is clinically relevant in light of recommendations for a ≥3% weight reduction to mitigate obesity‐related risks in East Asian populations.^30^ Considering that the intervention consisted of only four sessions, these findings suggest that even low‐intensity, mindfulness‐based approaches may offer meaningful benefits. Previous studies have reported average weight loss of 3%–5% with high‐intensity behavioral programs (e.g., 14+ sessions),^7^, ^31^ approximately 10% with pharmacotherapy, and up to 25% with bariatric surgery.^32^ MBIs, particularly those rooted in 3wCBT, have typically shown reductions of 3%–4% over 3–6 months.^23^ Although the weight reduction observed in our study was somewhat lower, the simplicity of the MEAL program may enhance its scalability and cost‐effectiveness. The intervention's setting in an occupational context suggests its ecological validity.

Shorter, group‐based weight loss interventions tend to attain higher completion rates than their longer counterparts.^33^ Consistent with this pattern, our four‐session MEAL program achieved a 100% completion rate, surpassing the typical 75%–80% observed in many multi‐month group‐based programs.^34^, ^35^ Notably, even MBIs, despite their psychological focus, have reported completion rates as low as 50% when extended to 6 months.^36^ The MEAL program yielded a meaningful reduction in baseline weight by 1.8%, aligning with findings that most weight loss in longer programs tends to occur within the first 3–6 months.^33^ Taken together, these results suggest that a brief, four‐session format may optimize adherence without compromising effectiveness, offering a practical and efficient solution for time‐constrained or high‐risk populations.

In the present study, all participants completed the MEAL program without dropout, indicating favorable feasibility and participant engagement despite the small sample size. Several structural and contextual factors may have contributed to this outcome. The MEAL program was implemented as a four‐session, workplace‐based intervention led by a psychiatrist trained in MBIs, who also served as the company's occupational health physician. This structure may have provided participants with a sense of accessibility and consistency throughout the program. Furthermore, the brief format prioritized experiential mindfulness practice over didactic instruction, emphasizing self‐awareness and practical behavioral application rather than prescriptive guidance. These characteristics may have supported continuous participation and adherence. By cultivating mindful awareness of eating‐related experiences and disrupting automatic or habitual eating patterns, the MEAL program emphasized “quality over quantity,” encouraging participants to derive satisfaction from the sensory and emotional aspects of food rather than the amount consumed. This approach likely enhanced participant engagement and adherence, thereby contributing to the favorable adherence observed in this pilot study.

Several limitations need to be noted. First, the sample size was small, limiting statistical power and generalizability. Second, the absence of a control group in this preliminary report restricts causal inference. Third, outcomes were limited to body weight; no psychological or behavioral mediators were assessed. Because this study retrospectively analyzed data obtained from routine occupational health activities, the inclusion of psychological or behavioral assessments was not feasible. Future prospective studies should incorporate validated measures of psychological flexibility, emotional regulation, and eating behaviors to clarify the mechanisms underlying MBIs. Fourth, selection bias may have occurred, as participants were self‐selected and potentially more motivated. Finally, this analysis only evaluated short‐term changes and did not assess the long‐term maintenance of weight loss, which limits conclusions about sustained effects.

The MEAL program shows promise as a pragmatic, MBI for weight management in occupational settings, although the preliminary findings have to be confirmed in further investigations, including randomized‐controlled trials with larger samples, extended follow‐up, and validated measures of mindfulness, self‐regulation, and health behavior change.

## AUTHOR CONTRIBUTIONS

Daisuke Yamaichi wrote the first draft of the manuscript. Daisuke Yamaichi and Kiwako Yokoyama conceived the study and contributed to study design, data collection, analysis, and interpretation. Hiroyuki Uchida reviewed and edited the manuscript. Daisuke Fujisawa contributed to study design, analysis, and interpretation, and supervised the study. All authors reviewed and approved the final manuscript.

## CONFLICT OF INTEREST STATEMENT

The authors declare no conflicts of interest.

## ETHICS APPROVAL STATEMENT

The study was approved by the Ethics Committee of Keio University (20190099).

## PATIENT CONSENT STATEMENT

The clinical trial was described in detail to the subjects, all of whom provided written informed consent.

## CLINICAL TRIAL REGISTRATION

This study is not registered because it is a retrospective record of medical practice.

## Data Availability

The data supporting this study are available from the corresponding author upon reasonable request.
